# Depression and distress in pediatric type 1 diabetes: a Latin American multicenter study

**DOI:** 10.1530/EC-26-0512

**Published:** 2026-08-31

**Authors:** Valeria Hirschler, Claudio D Gonzalez, Amanda Benitez, Andrea Escalante Marassi, Angela Figueroa Sobrero, Ernesto Bogado, Adriana Roussos, Laura Gaete, Angeles Arrigo, Florencia Grabois, Eugenia Andres, Maria Laura Arzamendia, Juan Pablo Rojas Godoy, Ethel Codner, Luciana Araujo, Luis F Palacios Porta, Claudia Molinari, Daniel R Witte

**Affiliations:** ^1^ Diabetes Argentine Society, Epidemiology, Buenos Aires, Argentina; ^2^ CEMIC, Pharmacology, Buenos Aires, Argentina; ^3^ Hospital Pediatrico Juan Pablo Segundo, Corrientes, Argentina; ^4^ Hospital Pediatrico Fernando Barreyro, Posadas, Misiones, Argentina; ^5^ Hospital San Roque, Parana, Entre Rios, Argentina; ^6^ Hospital de niños Ricardo Gutierrez, Buenos Aires, Argentina; ^7^ Hospital Prof. Ramon Exeni, Buenos Aires, Argentina; ^8^ Hospital Provincial Neuquén Dr Castro Rendon, Neuquén, Argentina; ^9^ Hospital de Niños Pedro de Elizalde, Buenos Aires, Argentina; ^10^ Hospital Clínico San Borja-Arriaran/IDIMI, Santiago de Chile, Chile; ^11^ Hospital Pediatrico Avelino Castelan, Resistencia, Chaco, Argentina; ^12^ Hospital Nacional A.Posadas, Buenos Aires, Argentina; ^13^ University of Buenos Aires, School of Pharmacy and Biochemistry, Mathematics, Buenos Aires, Argentina; ^14^ Steno Diabetes Center Aarhus, Aarhus University Hospital, Aarhus, Denmark

**Keywords:** Latin American children, type 1 diabetes, depression, distress

## Abstract

**Objective:**

To determine i) the prevalence of depressive symptoms and diabetes-related distress and ii) the association between their combined presence and metabolic outcomes in Latin American children with type 1 diabetes mellitus (T1DM).

**Methods:**

This cross-sectional study included children with T1DM of > 1 year duration from 10 diabetes centers in Argentina and Chile. Age, sex, socioeconomic status (SES), and HbA1c were evaluated. Depressive symptoms were assessed using the Center for Epidemiologic Studies Depression Scale (CES-D; range: 0–60; depression: ≥16), and diabetes distress was assessed using the Problem Areas in Diabetes (PAID; range: 0–80; distress: ≥40). Spearman correlation, phi coefficient, and multivariable logistic regression were used for statistical analysis.

**Results:**

Among 244 children (50% female), the median age was 13.4 years (IQR: 11.3–15.0) and the median HbA1c was 8.3% (IQR: 7.2–9.5). Depressive symptoms were present in 52% of participants, and diabetes distress was present in 26.6%. The agreement between CES-D and PAID categorical thresholds was fair (phi = 0.39). The Both-1 group (*n* = 55; 22.5%) (high CES-D and PAID) showed an inverse Spearman correlation with SES (*r* = −0.32, *P* < 0.01) and a positive correlation with HbA1c (*r* = 0.30, *P* < 0.01). In multivariable logistic regression, higher HbA1c levels (OR: 1.34; 95% CI: 1.1–1.6) and lower SES (OR: 0.36; 95% CI: 0.2–0.6) were independently associated with the Both-1 group (high CES-D and PAID), adjusted for age, sex, and BMI.

**Conclusion:**

In Latin American children with T1DM, the combined presence of depressive symptoms and diabetes-related distress was associated with poorer metabolic control and lower SES.

## Background

Managing type 1 diabetes (T1DM) entails complex and demanding daily tasks that can produce considerable emotional and psychosocial distress for children and their caregivers. Depressive symptoms are strongly associated with diabetes-related distress (1). Depression and diabetes-related distress may create major barriers to diabetes self-management, giving rise to poor metabolic control and quality of life. Understanding how these emotional factors interact is key for developing individualized care strategies that improve both psychological and metabolic outcomes (2).

Diabetes distress, defined as the psychological strain and frustration associated with living with and managing diabetes, has been strongly linked to poorer metabolic control, inadequate self-care, and elevated depressive symptoms (2). Although diabetes-related distress and depressive symptoms are strongly correlated, they denote distinct psychological entities. Accordingly, their assessment in clinical practice requires different screening tools, as depression scales assess broader affective and cognitive symptoms. In contrast, diabetes-specific instruments, such as PAID, assess diabetes-related emotional distress. A previous study in adults evaluating the convergent and divergent validity of the PAID questionnaire showed moderate-to-strong correlations with depressive symptoms and divergent validity with general health status, supporting its purpose as a diabetes-specific measure of emotional distress and suggesting that it should not be considered interchangeable with depression scales (3). Nevertheless, depressive symptoms in people with diabetes may sometimes reflect diabetes-specific distress rather than a distinct psychiatric disorder (4). Burns *et al.* suggested that diabetes distress and depressive symptoms may have a bidirectional association, with each possibly strengthening the other (1). This relationship may be particularly relevant because diabetes distress arises from the daily challenges of managing the disease (5). Importantly, when both conditions are analyzed together, associations between depressive symptoms and metabolic outcomes often disappear once diabetes distress is considered, suggesting that distress may play a more central role in these relationships (6).

Although depression and distress are frequent among individuals with diabetes, most studies have focused on adults, with limited research in children and adolescents with T1DM (2, 3, 4, 5, 6, 7). As far as we know, no previous studies have examined the relationship between depressive symptoms and diabetes-related distress in children or adolescents with T1DM in Latin America. The objective of this study was to determine i) the prevalence of depressive symptoms and diabetes-related distress and ii) the association between the combined presence of depression and distress and relevant metabolic outcomes in Latin American children with T1DM.

## Methods

### Study design and participants

An observational, multicenter, cross-sectional study was conducted between January and August 2025 across 10 pediatric diabetes units in Argentina and Chile. All eligible patients attending routine visits were invited to participate. All 10 participating centers were referral institutions in Latin America that provided specialized care for children with T1DM. These centers either were exclusively pediatric or served as pediatric referral hospitals within public or academic healthcare systems. Most patients attending these centers belong to low- or middle-income households. To account for potential regional differences, centers were grouped into four categories based on geographic location, demographic characteristics, and urbanization: rural provinces (*n* = 96; 39.3%; Chaco, Corrientes, and Misiones, Argentina), Neuquén (*n* = 22; 9.0%; southern region, Argentina), urban 1 (*n* = 100; 41.0%; Buenos Aires city and suburbs, Argentina), and urban 2 (*n* = 26; 10.7%; Santiago, Chile). Buenos Aires and Santiago differ substantially in population density and infrastructure – approximately 15.6 million vs 7.1 million inhabitants – factors that may influence healthcare access and the complexity of diabetes care delivery.

### Eligibility criteria

Inclusion criteria were children aged 8–17 years with a confirmed diagnosis of T1DM for more than one year. Screening was initiated at the age of 8 years, as children at this developmental stage generally have the cognitive and language abilities required to articulate emotional experiences, such as distress (8). Moreover, families of school-age children generally begin sharing diabetes management responsibilities, and higher levels of distress in either children or parents may hinder effective co-management (8). Exclusion criteria included celiac disease, genetic syndromes, intellectual disabilities, and polyendocrinopathies.

### Informed consent

Written informed consent was obtained from parents or caregivers, and assent was received from all children. Data were pseudonymized using coded identifiers to ensure confidentiality. The study adhered to the Declaration of Helsinki and national research ethics standards.

### Clinical and sociodemographic variables

Sociodemographic and clinical variables included age, sex, diabetes duration, parental education, school type (public or private), diabetic ketoacidosis (DKA) episodes in the past year, body mass index (BMI), use of continuous glucose monitoring (CGM), insulin pump therapy, presence of microvascular complications, and daily insulin dose per kilogram. Anthropometric measurements followed standardized procedures. BMI was calculated as weight (kg)/height^2^ (m^2^), and age- and sex-specific z-scores were derived from CDC reference data. Metabolic control was assessed using the most recent HbA1c measurement and the HbA1c measurement obtained approximately one year earlier (year 2), both measured by high-performance liquid chromatography. The year 2 HbA1c value was obtained retrospectively from the medical records.

Given the heterogeneity of healthcare systems across Latin American countries, traditional indicators, such as public versus private healthcare coverage, are not directly comparable. Furthermore, parental occupation was not included due to the high prevalence of informal employment in Argentina and the potential for underreporting, which may compromise data accuracy. Therefore, a composite variable was constructed to better capture socioeconomic status (SES). In this context, socioeducational indicators may serve as a useful proxy for SES in Latin American pediatric populations.

This variable combined the child’s school type (public vs private) and parental educational attainment (high school completion or higher vs less than high school). It was categorized into three levels: i) children attending public school with parents who had not completed high school, ii) intermediate category, and iii) children attending private school with parents who had completed high school or higher.

### Assessment of depressive symptoms and diabetes distress

Depressive symptoms were assessed using the Center for Epidemiologic Studies Depression Scale (CES-D) (9). The CES-D contains 20 items rated on a four-point Likert scale (0 = rarely/never to 3 = most of the time) (9). Total scores range from 0 to 60, with higher scores indicating greater depressive symptomatology; scores ≥16 denote clinically significant symptoms (10).

Diabetes-related distress was measured using the Problem Areas in Diabetes – Pediatric Version (PAID), administered by trained healthcare professionals during clinic visits (11). The validated Spanish version was used, consisting of 20 items rated from 0 (‘no problem’) to 4 (‘serious problem’), with total scores categorized as low (0–19), moderate (20–39), or high (40–80) distress (12). Higher PAID scores reflect a greater emotional burden associated with diabetes management and may indicate the need for psychological or multidisciplinary intervention.

### Definition of study groups

Participants were categorized into four groups according to the combined presence or absence of CES-D depressive symptoms (≥16) and PAID diabetes distress (≥40). For each score, a value of ‘1’ indicated that the participant was above the clinical cutoff (presence of symptoms) and a value of ‘0’ indicated that the participant was below the cutoff (absence of symptoms). The four resulting groups were as follows:

- Both-0: PAID = 0 and CES-D = 0 – children without diabetes distress and without depressive symptoms.
- Both-1: PAID = 1 and CES-D = 1 – children with both high diabetes distress and depressive symptoms.
- PAID-only: PAID = 1 and CES-D = 0 – children with high diabetes distress but without depressive symptoms.
- CES-D-only: PAID = 0 and CES-D = 1 – children with depressive symptoms but without diabetes distress.

### Data analysis

Data were analyzed using SPSS, version 22. The distribution of continuous variables was assessed using the Shapiro–Wilk test. Variables with non-normal distributions were presented as median (interquartile range (IQR)), whereas categorical variables were presented as frequencies and percentages. Comparisons between two groups were made using the Mann–Whitney U test, and comparisons among more than two groups were made using the Kruskal–Wallis test. When overall group differences were significant, post hoc pairwise comparisons were made using the Dunn–Bonferroni correction. Categorical variables were compared using the chi-square test or Fisher’s exact test, as appropriate. Comparisons of clinical and sociodemographic characteristics were made across the four predefined study groups (Both-0, Both-1, PAID-only, and CES-D-only). Univariate Spearman correlation analyses were performed to evaluate the associations between the Both-1 group and the most recent HbA1c, HbA1c at year 2, SES, DKA episodes in the previous year, age, sex, and BMI. A multivariable logistic regression analysis was performed using the combined presence of depressive symptoms and high diabetes-related distress (Both-1 group: CES-D = 1 and PAID = 1) as the dependent variable. Independent variables included in the multivariable model were selected based on clinical relevance and statistically significant associations identified in the univariate analyses. Statistical significance was defined as *P* < 0.05.

## Results

### Participant characteristics

Initially, 255 children and adolescents were assessed; 11 were excluded due to missing data or intellectual disability, leaving 244 participants (122 females, 50%) in the final analysis (4.3% excluded; 95.7% included). The median age was 13.4 years (IQR: 11.4–15.7), and the median duration of diabetes was 4.7 years (IQR: 2.7–7.7). The prevalence of overweight or obesity was 30.3% (*n* = 74), the prevalence of nephropathy was 2.0% (*n* = 5), and the prevalence of retinopathy was 0.8% (*n* = 2). Only 23.4% (*n* = 57) had a most recent HbA1c value below the recommended threshold of 7%, and only 20.5% (*n* = 50) had their previous HbA1c value below this threshold. Table 1 summarizes the demographic and clinical characteristics of the participants by sex. Compared with males, females had a younger age at onset and higher PAID scores, BMI, and BMI percentile. The median PAID score was 28 (IQR: 16–41), with 26.6% (*n* = 65) experiencing high distress (PAID ≥ 40). The median CES-D score for the total sample was 15 (IQR: 8–25), with 52.0% (*n* = 127) scoring above the clinical threshold for depression.

**Table 1:** Clinical characteristics of the sample according to sex.

Variable	Males (*n* = 122)	Females (*n* = 122)	Total (*n* = 244)
Age (years)	13.45 (11.40–16.00)	13.36 (11.25–15.18)	13.40 (11.37–15.66)
Age at onset (years)*	8.57 (6.51–11.09)	7.59 (5.25–10.31)	8.09 (5.84–10.70)
T1DM duration (years)	4.63 (2.54–7.52)	4.78 (2.85–7.71)	4.71 (2.67–7.66)
Most recent HbA1c %	8.00 (7.20–9.00)	8.40 (7.20–9.70)	8.20 (7.20–9.20)
HbA1c Y2 (%)	8.00 (7.17–9.00)	8.25 (7.35–9.88)	8.00 (7.20–9.28)
Insulin dose (IU/kg/day)	0.82 (0.66–1.00)	0.92 (0.69–1.06)	0.88 (0.68–1.04)
BMI (kg/m^2^)*	19.75 (17.58–22.19)	21.32 (19.31–23.88)	20.78 (18.08–23.22)
BMI percentile*	64.36 (41.41–82.42)	78.34 (61.74–89.97)	71.39 (51.19–86.60)
CES-D score	14.00 (8.00–24.00)	16.50 (9.00–28.00)	15.00 (8.00–25.00)
PAID score*	24.00 (13.25–38.75)	30.00 (18.25–43.75)	28.00 (16.00–41.25)

T1DM, type 1 diabetes; Y, year; PAID, Problem Areas in Diabetes – Pediatric Version; CES-D, Center for Epidemiologic Studies Depression Scale. Data are presented as median (interquartile range (IQR)). Continuous variables were compared using the Mann–Whitney U test. Variables marked with an asterisk differed significantly between males and females (*P* < 0.05).

Regarding socioeconomic indicators, based on the composite SES variable, 42.6% (*n* = 104) were classified as low SES (public school and parents without high school completion), 36.9% (*n* = 90) were classified as intermediate SES, and 20.5% (*n* = 50) were classified as high SES (private school and parents with high school completion or higher).

### Concordance between CES-D and PAID scores

High diabetes distress (PAID ≥ 40) was present in 43.3% of children with depressive symptoms compared with 8.5% of those without depressive symptoms. Conversely, PAID scores below the clinical cutoff were observed in 91.5% of children without depressive symptoms and in 56.7% of those with depressive symptoms (Table 2).

**Table 2:** Cross-classification of depressive symptoms (CES-D) and diabetes distress (PAID) in 244 children with type 1 diabetes.

		Depressive symptoms (CES-D)		Total
		CES-D < 16	CES-D ≥ 16	
Distress (PAID)	PAID < 40	107	72	179
	PAID ≥ 40	10	55	65
Total		117	127	244

PAID, Problem Areas in Diabetes – Pediatric Version; CES-D, Center for Epidemiologic Studies Depression Scale. Data are presented as *n*.

The agreement between CES-D and PAID categorical thresholds was fair (phi = 0.39), indicating limited overlap between the two scores, as shown by the distribution of participants across the four quadrants in Fig. 1.

**Figure 1:**
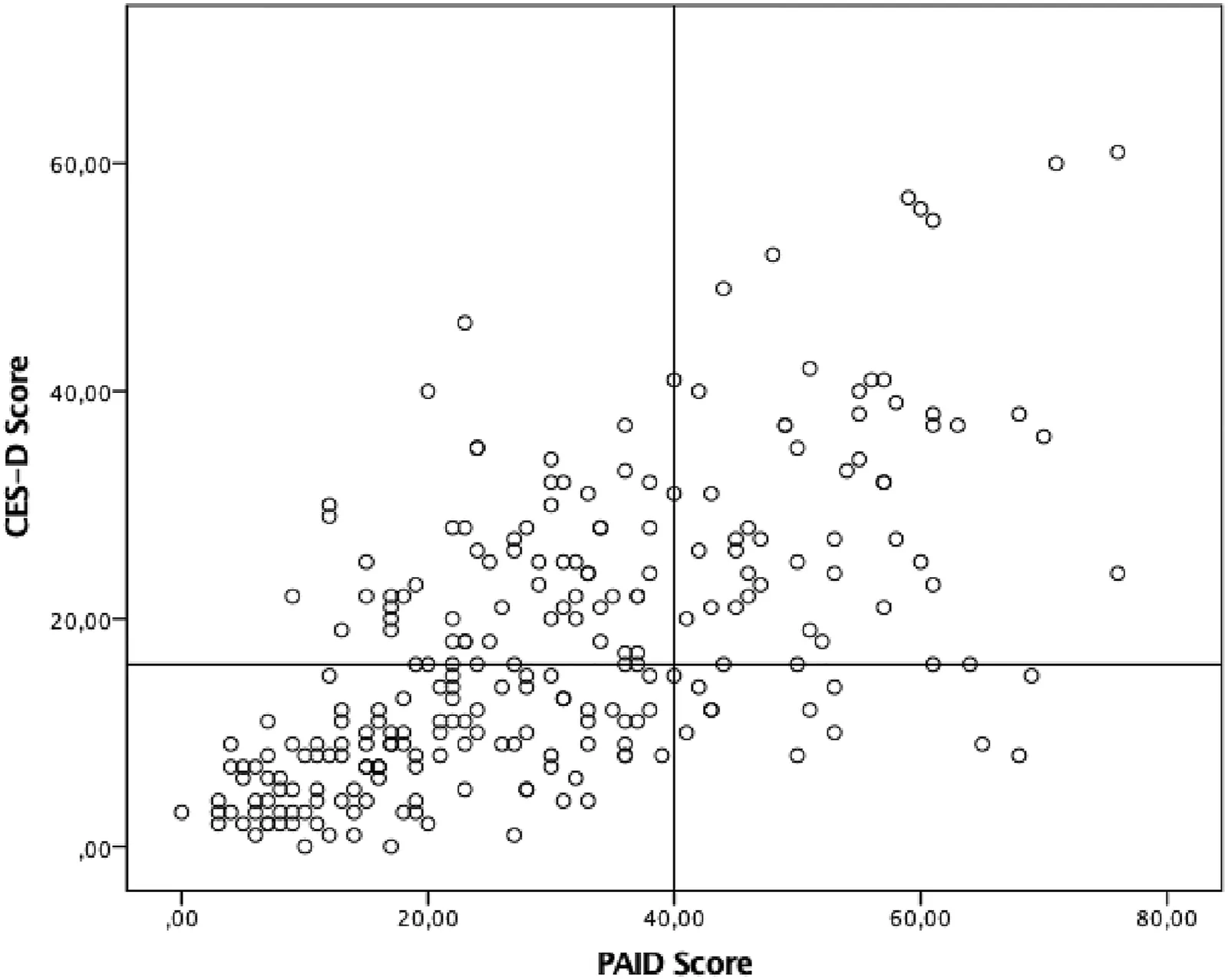
Scatterplot of PAID and CES-D scores. The vertical and horizontal lines indicate the clinical cutoff points. The upper-left quadrant represents participants with high CES-D scores and normal PAID scores (CES-D-only), the lower-right quadrant represents participants with high PAID scores and normal CES-D scores (PAID-only), the upper-right quadrant represents participants with high scores on both scales (Both-1), and the lower-left quadrant represents participants with normal PAID and normal CES-D scores (Both-0).

### Clinical and demographic differences across combined CES-D and PAID groups

Table 3 summarizes the clinical and demographic characteristics across the four predefined study groups (Both-0, Both-1, PAID-only, and CES-D-only). Age, diabetes duration, and insulin dose did not differ significantly between groups. Compared with the Both-1 group (PAID = 1 and CES-D = 1), the Both-0 group (PAID = 0 and CES-D = 0) had higher SES and lower median HbA1c values at the most recent assessment and at Y2. Post hoc pairwise comparisons using the Dunn–Bonferroni correction confirmed that the Both-1 group had significantly higher HbA1c values at the most recent assessment and at Y2, as well as significantly lower SES, than the Both-0 group. The prevalence of DKA was 4.7% in the Both-0 group, 21% in the Both-1 group, 20% in the PAID-only group, and 8.5% in the CES-D-only group, with a significant difference between groups (*P* = 0.015).

**Table 3:** Comparison of clinical and sociodemographic characteristics across PAID and CES-D categories.

Variable	Both-0 PAID = 0 & CES-D = 0	Both-1 PAID = 1 & CES-D = 1	PAID-only PAID = 1 & CES-D = 0	CES-D-only PAID = 0 & CES-D = 1
	*n* = 107	*n* = 55	*n* = 10	*n* = 72
Age (years)	13.23 (11.06–15.73)	13.19 (11.90–15.25)	13.31 (12.57–15.70)	13.53 (11.22–15.42)
T1DM duration (years)	4.60 (3.10–6.75)	4.26 (3.12–6.94)	6.09 (4.59–8.28)	4.32 (3.11–6.61)
High SES, *n* (%)*	28 (26.2)	3 (5.5)	1 (10.0)	18 (25.0)
Insulin dose (IU/kg/day)	0.83 (0.65–1.00)	0.93 (0.73–1.10)	0.82 (0.69–1.00)	0.92 (0.70–1.04)
Most recent HbA1c %*	7.70 (6.90–8.90)	9.00 (8.20–10.40)	8.40 (7.90–8.90)	8.20 (7.50–9.10)
HbA1c Y2 (%)*	7.50 (6.98–8.93)	9.00 (8.00–10.30)	8.15 (7.78–8.83)	8.35 (7.28–9.10)
BMI (kg/m^2^)	20.28 (17.78–22.93)	20.91 (18.38–23.55)	20.31 (18.22–22.46)	21.13 (18.78–23.65)
BMI percentile	69.96 (47.86–84.81)	71.99 (54.30–89.37)	70.53 (45.62–80.32)	78.31 (56.44–89.74)

PAID, Problem Areas in Diabetes – Pediatric Version; CES-D, Center for Epidemiologic Studies Depression Scale; SES, socioeconomic status; T1DM, type 1 diabetes mellitus; BMI, body mass index; HbA1c, glycated hemoglobin; Y, year. Data are presented as median (IQR) or *n* (%). Continuous variables were compared using the Kruskal–Wallis test and categorical variables using the chi-square test or Fisher’s exact test, as appropriate.

* *P* < 0.01.

Post hoc pairwise comparisons using the Dunn–Bonferroni correction showed that the Both-1 group had significantly higher HbA1c values (Y1 and Y2) and lower socioeconomic status (SES) than the Both-0 group.

### Associations between the Both-1 group and clinical and demographic characteristics

In univariate Spearman correlation analyses, the Both-1 group (high CES-D and high PAID scores) was significantly and inversely associated with SES (r = −0.32, *P* < 0.01) and positively associated with the most recent HbA1c (*r* = 0.30, *P* < 0.01), HbA1c at year 2 (*r* = 0.28, *P* < 0.01), and the number of DKA episodes in the past year (*r* = 0.19, *P* < 0.01). There were no significant associations with age, diabetes duration, or BMI.

Variables for the multivariable logistic regression analysis were selected based on clinical relevance and statistically significant associations identified in the univariate analyses. The analysis included the most recent HbA1c, socioeconomic status (SES), age, sex, and BMI. In the multivariable model, with membership in the Both-1 group as the dependent variable, the most recent HbA1c (OR: 1.34; 95% CI: 1.1–1.6) and SES (OR: 0.36; 95% CI: 0.2–0.6) remained independently associated with the outcome after adjustment for age, sex, and BMI (Table 4).

**Table 4:** Multiple logistic regression analysis.

	B	S.E.	Sig.	OR	95% CI for OR	
					Lower	Upper
Sex	−0.21	0.37	0.56	0.81	0.40	1.66
Age (year)	0.01	0.08	0.87	1.01	0.87	1.18
BMI	0.04	0.06	0.55	1.04	0.92	1.17
Most recent HbA1c %	0.29	0.10	<0.01	1.34	1.11	1.63
SES	−1.03	0.28	<0.01	0.36	0.21	0.62

Dependent variable: the Both-1 group (high PAID and high CES-D).

## Discussion

In this multicenter cohort of 244 Latin American children with T1DM, depressive symptoms and diabetes-related distress were frequent, and their combined presence was associated with poorer metabolic control and lower SES. However, the agreement between CES-D and PAID categorical thresholds was fair (phi = 0.39), indicating limited overlap between the two scores. To the best of our knowledge, this is the first study to explore the association between depressive symptoms and diabetes-related distress in Latin American children. Further prospective studies are needed to confirm these findings and clarify their clinical implications.

More than half of the participants (52.0%) scored above the CES-D threshold, while 26.6% experienced high diabetes distress. The prevalence of depressive symptoms was higher than that reported in earlier studies of adolescents with T1DM, which ranged between 21 and 41.7% (13, 14). Previous reports have shown a bidirectional association between depressive symptoms and diabetes-related distress (15). Accordingly, in a prospective study, Burns *et al.* demonstrated a reciprocal association between depressive symptoms and diabetes distress, with each predicting increases in the other over time (1). In contrast, the Dimensions of Care for Diabetes Study reported that nearly one-third of patients with diabetes who screened positive for depression did not have elevated distress (16). This distinction indicates that depression and diabetes distress, while correlated, are partially independent psychological reactions requiring different clinical approaches. Consistently, we found that although 43.3% of children with depressive symptoms exhibited high diabetes distress, more than half did not. Furthermore, the agreement between the two scores was only fair (phi = 0.39), indicating limited overlap and suggesting that both instruments are needed to identify children requiring support. This partial overlap may reflect that CES-D captures general depressive symptoms, whereas PAID reflects diabetes-specific emotional burden. From a clinical perspective, different combinations of PAID and CES-D scores may identify children with distinct support needs. Children with high PAID but low CES-D scores may benefit primarily from diabetes-specific interventions intended to reduce treatment burden, whereas those with high CES-D but low PAID scores may call for further psychological evaluation for depressive symptoms beyond diabetes-related distress.

Children in the Both-1 group may represent a particularly vulnerable subgroup who could benefit from integrated psychological and diabetes care interventions.

The continuous and demanding nature of diabetes management is a major source of distress. Current guidelines from the American Diabetes Association recommend that clinicians routinely evaluate both depressive symptoms and diabetes distress to enhance self-management and quality of life (17). A prospective study in adults with T1DM suggests that diabetes-related distress, more than clinical depression itself, may be the primary driver of suboptimal self-care and poor metabolic outcomes (18). Similarly, in adults with type 2 diabetes, when both distress and depressive symptoms were evaluated together, the association between depression and glycemic control often became nonsignificant once distress was included in the model (6), indicating that symptom-based depression scales may capture diabetes-related emotional burden instead of a distinct psychological disorder. Ehrmann and colleagues demonstrated that higher diabetes-related distress predicted increased depressive symptoms six months later, while baseline depressive symptoms similarly forecasted greater distress over time, even after accounting for initial scores (15).

Regarding the second study objective, the Both-1 group was significantly associated with higher HbA1c levels and lower SES in both univariate analysis and the multivariable logistic regression model. Children exhibiting both high diabetes-related distress and depressive symptoms had significantly higher HbA1c values than those with neither condition, supporting an association between the combined psychological burden and poorer glycemic control. Children with lower SES had significantly higher combined CES-D and PAID scores, whereas higher SES was associated with approximately 65% lower odds of presenting with combined elevated CES-D and PAID scores after adjustment for age, sex, and BMI. These data highlight the importance of social and economic factors in the psychosocial burden of pediatric T1DM and suggest that children from more disadvantaged backgrounds may require additional psychological support and focused interventions (19, 20). Individuals with T1DM must constantly adapt to variable treatment demands and glycemic variability, which can alter perceived stress and self-efficacy (5). Reducing distress has been shown to improve depressive symptoms and general well-being, stressing the value of targeted psychosocial interventions (21). Early identification and systematic screening for both depressive symptoms and diabetes-related distress should therefore be a routine aspect of comprehensive diabetes care (5, 22).

### Strengths and limitations

This study has several strengths, notably its multicenter design and the combined assessment of CES-D and PAID, which underscores the value of using both instruments in pediatric diabetes care. Despite these strengths, several limitations should be acknowledged. First, given the heterogeneity of healthcare systems across Latin American countries, traditional indicators, such as public versus private healthcare coverage, are not directly comparable. Notably, parental occupation was not included due to the high prevalence of informal employment in Argentina and the potential for underreporting, which may compromise data accuracy. Therefore, we constructed a composite SES variable combining parental education and school type, which may better capture socioeconomic conditions in this regional context. Second, the use of a fixed cutoff to define elevated depression and distress may have reduced precision and underestimated gradations of severity.

## Conclusion

In Latin American children with T1DM, the combined presence of depressive symptoms and diabetes-related distress was associated with poorer metabolic control and lower SES. Using both instruments may be important for fully identifying children who may require support. Further prospective studies should be performed to confirm the results.

## Declaration of interest

The authors declare that there is no conflict of interest that could be perceived as prejudicing the impartiality of the research reported.

## Funding

This research received no specific grant from any funding agency in the public, commercial, or not-for-profit sectors.

## Author contribution statement

VH conceived and designed the study, supervised data collection, and wrote the manuscript. CM performed the statistical analysis and contributed to data interpretation. CDG contributed to study design and methodology. DRW contributed to data interpretation and critically revised the manuscript. AB, AEM, AFS, EB, AR, LG, AA, FG, EA, MLA, JPRG, EC, LA, and LFPP contributed to patient recruitment and data collection across participating centers. All authors reviewed and approved the final version of the manuscript.

## Ethics approval

Ethical approval for this multicenter study was obtained under approval numbers PRIISA BA 14851 and PRIISA BA 15594. Written informed consent was obtained from parents or caregivers, and assent was obtained from all participating children.
